# Bilateral Basal Ganglia Infarction After Intranasal Use of Cocaine: A Case Report

**DOI:** 10.7759/cureus.4405

**Published:** 2019-04-08

**Authors:** Oscar Cisneros, Katherine Garcia de de Jesus, Eric O Then, Razia Rehmani

**Affiliations:** 1 Internal Medicine, St. Barnabas Hospital Health System / Albert Einstein College of Medicine, Bronx, USA; 2 Gastroenterology, St. Barnabas Hospital Health System / Albert Einstein College of Medicine, Bronx, USA; 3 Neuroradiology, St. Barnabas Hospital Health System / Albert Einstein College of Medicine, Bronx, USA

**Keywords:** cocaine, bilateral basal ganglia, ischemic infarct, mri

## Abstract

We present a case of a young man who developed bilateral basal ganglia infarct after intranasal use of cocaine. Cerebral ischemic infarcts are a known complication of cocaine use. This complication is rare and has been reported in the past with cocaine and concomitant use of other drugs such as heroin and amphetamines.

## Introduction

Cocaine is an alkaloid extracted from leaves that can be chewed or be prepared in concentrated forms, the most common of which is cocaine hydrochloride. Historically the neurologic complications of cocaine increased after the introduction of crack in the eighties [1,2]. Crack cocaine is smokable and achieves higher central nervous system (CNS) levels in less time than intranasal administration of cocaine. Several studies have demonstrated the association between cerebrovascular disease and the use of cocaine [3-10]. In the CNS, cocaine can cause a variety of complications such as stroke, vasculitis, seizures, cognitive impairment, parenchymal and subarachnoid hemorrhage, and toxic encephalopathy [1-5,11-15]. Given the wide scope of these complications, radiologists should become familiar with the radiologic findings that are associated with cocaine ingestion.

## Case presentation

A 40-year-old Hispanic man with a past medical history of human immunodeficiency virus (HIV) was brought to the emergency department complaining of right upper extremity (RUE) weakness and numbness for four days with associated bitemporal headache and generalized fatigue. The patient reported first time use of intranasal cocaine and heroin, after which he lost consciousness and woke up approximately four hours later with new onset RUE and headache. His cluster of differentiation 4 (CD-4) count was reported above 500 cells/mm3 and viral load (VL) was undetectable. The patient did not have any known CNS complications in the past.

**Figure 1:**
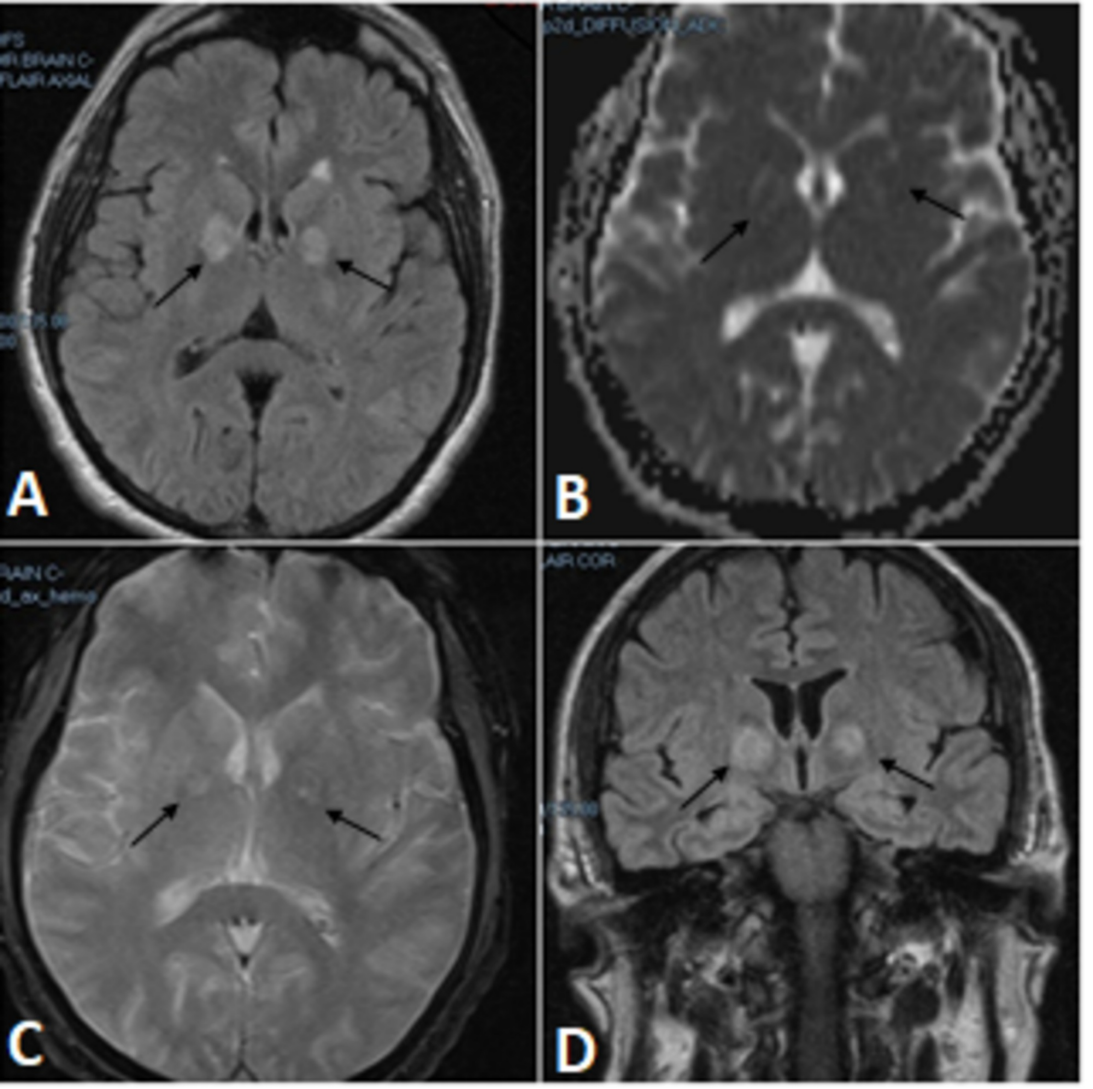
Magnetic resonance imaging (MRI) of the brain. MRI of the brain revealed two areas of increased T2/FLAIR signal within the medial aspect of both basal ganglia, measuring 16 mm in the right and 12 mm on the left involving each globus pallidus and the genu of the internal capsule.

**Figure 2:**
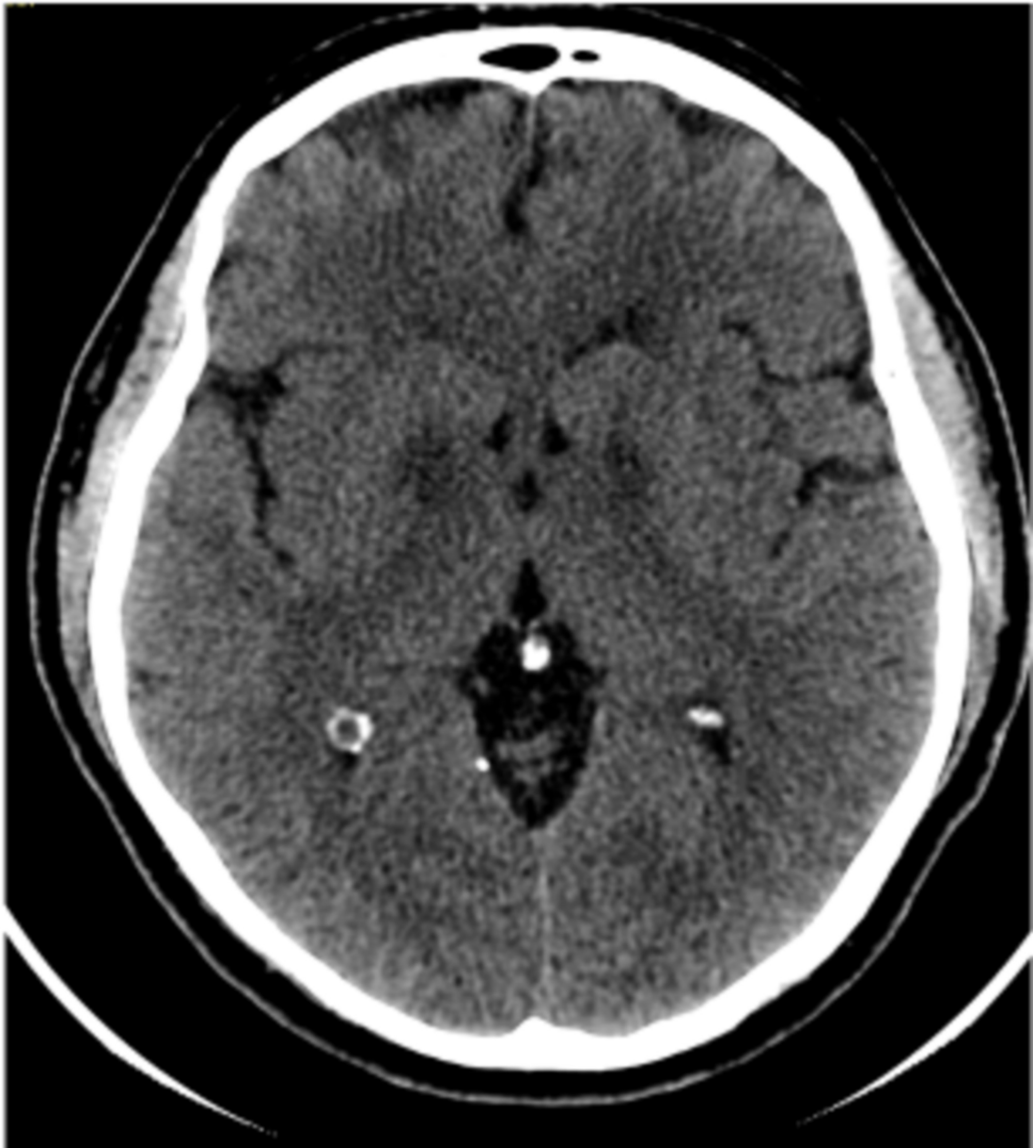
Computed tomography (CT) of the brain. Normal CT of the brain.

On physical examination, his blood pressure was 151/97 mm Hg and pulse was 82 and regular. He was alert and cooperative. His cranial nerves were intact. His motor exam, however, was abnormal in the RUE with 3/5 arm strength and wrist drop; the strength and tone of the other extremities were normal throughout. Deep tendon reflexes were normal bilaterally, but his gait could not be evaluated. His sensory function decreased to pin sensation at the RUE and normal sensation was noted in the rest of the extremities and face. Laboratory testing was normal except for an elevated creatinine of 6.9 mg/dl, creatine phosphokinase (CPK) of 7855 IU/l, alanine transaminase (ALT) of 139 IU/l, and aspartate transaminase (AST) of 109 IU/l. Urine toxicology was positive for metabolites of cocaine and heroin. Magnetic resonance imaging (MRI) of the brain was done and it revealed two areas of increased T2/FLAIR signal within the medial aspect of both basal ganglia, measuring 16 mm in the right and 12 mm on the left involving each globus pallidus and the genu of the internal capsule, as can be seen in Figures 1A-1D. His chest radiography was normal, computerized tomography (CT) of the brain, as can be seen in Figure 2, and cervical spine were normal. His electrocardiogram was normal.

In the subsequent days, his kidney function and rhabdomyolysis improved. The patient remained fully awake, alert and oriented, but the weakness of his RUE persisted. The patient decided to leave against medical advice despite full explanation of the risk of leaving.

The patient was contacted over the phone and he informed us that he followed up with his primary care physician and reported improvement of the weakness. He received physical therapy and was independent in all activities of daily living and functional mobility. His only limitation was a moderate decrease in fine motor coordination of the RUE.

## Discussion

Cocaine use has become a widespread problem is the United States of America. In a survey done by Substance Abuse and Mental Health Services administration (SAMHSA), 2.4% of the general population reported cocaine use in the previous month [16]. Bilateral lesions in ischemic infarcts of the basal ganglia are rare. Conditions known to be associated with bilateral basal ganglia infarct include carbon monoxide poisoning, methanol intoxication, diffuse hypoxic or ischemic injury from cardiorespiratory arrest, hypovolemia due to trauma, bibasilar artery occlusion, and intravenous heroin use [9].

Cocaine use, even in first-time users, can cause a wide variety of pathologies. Our patient represents an unusual case because of the symmetrical bilateral ischemic lesions involving the globus pallidus and internal capsule. The mechanisms by which cocaine produces cerebrovascular damage are multifaceted [4]. Various mechanisms are believed to contribute to the increased risk of ischemic stroke associated with cocaine. Cerebral vasospasm secondary to cocaine ingestion may affect large cranial arteries as well as the cortical microvasculature. Cocaine also increases the risk of vascular thrombosis by potentiating platelet aggregation [4,5,10,12]. The mechanism by which heroin may cause bilateral basal infarctions is through hypoxia from hypoventilation and hypotension [9]. In our case the patient lost consciousness for several hours. This might have been another contributing mechanism of the lesion. MRI imaging in our patient showed high fluid attenuation inversion recovery (FLAIR) signal changes bilaterally in the basal ganglia, globus pallidus, and internal capsule, as can be seen in Figures 1A-1D. None of these changes were appreciable on the CT scan of the brain performed earlier on our patient, as can be seen in Figure 2.

Recently newer imaging modalities have emerged that can demonstrate functional status in addition to structural lesions, unlike CT or MRI that only demonstrate structural lesions. However these newer, functional neuroimaging techniques such as single photon emission computed tomography (SPECT) and positron emission tomography (PET) may demonstrate significant changes in brain functioning even in the absence of structural lesions [1,9,13]. In a study involving patients with chronic cocaine use, MRI data demonstrated that these patients (without clinically apparent cerebrovascular symptoms or major risk factors) suffered from accelerated cerebrovascular damage when compared to age-matched normal control subjects. The lesions described in this study, however, were more common in the white matter of the cerebral hemispheres and insular region as opposed the unusual lesions found in our patient [10]. A dynamic susceptibility contrast MRI study also demonstrated that global cerebral blood volume (CBV) reduction is observed following intravenous (IV) cocaine administration. The study failed to show dose-effect relationship likely due to single time point measurement CBV and cocaine dosage [7]. Diffuse vasoconstriction or decrease in CBV is clearly associated with cerebrovascular events in animals and humans; however, this could not explain the findings in our case. There is one reported case of a young woman who developed vasculitis of the small arteries of the deep white matter and basal ganglia bilaterally. The cause was later confirmed by biopsy and is the only case to our knowledge of vasculitis due to cocaine [3]. This could represent a specific mechanism by which our patient developed the bilateral lesions; however, we did not perform a biopsy to confirm this theory. We highlight the additive role of cocaine in the context of CNS ischemia and the relevance of using neuroimaging as an accurate diagnostic tool in substance abuse patients with new neurologic findings on physical exam.

## Conclusions

This case is an uncommon one since it represents an ischemic event in the context of cocaine and heroin abuse that exhibited clinical manifestations of new onset extremity weakness and numbness with an affectation of bilateral basal ganglia in neuroimaging. Basal ganglia infarct is a rare condition that can be due to a variety of causes. Radiologists and neurologists must be aware of this entity and its most common etiologies as it might represent serious permanent complications for afflicted patients. CT and MRI are very useful in diagnosing this condition.
